# Structural basis for a central permeation pathway in the P2X1 receptor

**DOI:** 10.1038/s41421-026-00881-w

**Published:** 2026-06-04

**Authors:** Heng Zhang, Pengpeng Wu, Zhiyong Gu, Youwei Xu, Wen Hu, Qingning Yuan, Bingqing Xia, H. Eric Xu, Zhaobing Gao

**Affiliations:** 1 The State Key Laboratory of Drug Research, Shanghai Institute of Materia Medica, Chinese Academy of Sciences, Shanghai, China; 2 University of Chinese Academy of Sciences, Beijing, China; 3 The Shanghai Advanced Electron Microscope Center, Shanghai Institute of Materia Medica, Chinese Academy of Sciences, Shanghai, China

**Keywords:** Cryoelectron microscopy, Calcium signalling

## Abstract

The ion permeation pathway is a critical determinant of ion channel function and selectivity; however, the structural basis for ion permeation in the P2X1 receptor, an ATP-gated ion channel crucial for platelet activation, thrombosis, and male infertility, remains incompletely understood. Here, we present high-resolution cryo-electron microscopy (cryo-EM) structures of the P2X1 receptor, which reveal a central ion permeation pathway spanning the entire extracellular domain, complementing the existing paradigms of ion channel architecture for the P2X receptor family. Within this pathway, we identify specific sites that coordinate hydrated calcium ions, including an aspartate ring that acts as a selectivity filter at the apex of the central vestibule. We also discover that a small molecule, 3,5-bis(trifluoromethyl)aniline, binds at the top of the central vestibule and potently inhibits cation flux through this central permeation pathway. Our findings reveal a new inhibitor-binding site in the P2X1 receptor. These insights provide a structural framework for the rational design of subtype-specific P2X receptor inhibitors targeting the central vestibule.

## Introduction

The ion permeation pathway is essential for the function and specificity of ion channels, as it influences how ions traverse and interact within these critical cellular gateways^1–6^. Among the seven ATP-gated cation channels in the P2X receptor family, the P2X1 receptor is distinguished by its unique physiological and pathological roles^7,8^. Predominantly expressed in neutrophils, thymocytes^9^, platelets^10,11^, and smooth muscle cells^12,13^, the P2X1 receptor is known for its rapid desensitization^14,15^ and pronounced preference for calcium influx at low extracellular ATP concentrations (< 1 μM)^16^. Notably, this specificity underlies the involvement of the P2X1 receptor in critical processes such as platelet aggregation, hemostasis, and wound healing. Although the role of the P2X1 receptor in platelet aggregation is secondary to that of the P2Y12 receptor, the P2X1 receptor is associated with a reduced bleeding risk, positioning it as a potential target for therapeutic intervention^17–20^.

The P2X receptor family comprises seven members, P2X1–7, which assemble as homotrimeric or heterotrimeric cation channels. Like the other six members of this family, the P2X1 receptor features a symmetric extracellular domain. Its ion permeation pathway includes narrow transmembrane pores that are possibly connected either to the central symmetrical pathways in the extracellular domain or to lateral fenestrations^21,22^. However, the roles of these potential routes, particularly the central pathway versus the lateral fenestrations, in ion permeation are not well understood. Although lateral fenestrations have traditionally been considered the primary permeation path in P2X receptors^1,23^, their specific roles in cation dehydration and selectivity remain incompletely defined.

Among its various physiological roles, the P2X1 receptor mediates the influx of calcium ions in response to extracellular ATP, thereby promoting cellular processes that are crucial for hemostasis and tissue repair^10,24,25^. Additionally, the P2X1 receptor plays a role in sustaining the activity of leukemia-initiating cells, and the use of P2X1 receptor antagonists effectively inhibits the proliferation of acute myeloid leukemia cells^26^. Nevertheless, the exploration of therapeutics targeting the P2X1 receptor is still in its early stages^17^, and the identification of new inhibitor-binding sites, together with the discovery of novel inhibitors, remains important and largely unexplored.

To address these gaps, we determined the structures of the P2X1 receptor at different calcium concentrations and integrated the structural data with electrophysiological data to investigate the central ion permeation pathway and its calcium-selective sites. Following the identification of a crucial calcium-selective site at the apex of the central vestibule, we conducted targeted virtual screening and validated the efficacy of 3,5-bis(trifluoromethyl)aniline (BTFA) as an effective inhibitor using cryo-electron microscopy (cryo-EM) and electrophysiology. BTFA binding at the central vestibule effectively inhibits cation permeation, supporting the involvement of the central vestibule in ion conduction. This binding site may also pave the way for the rational design of subtype-specific P2X receptor inhibitors for treating patients with thrombosis and inflammatory diseases.

## Results

### Structural determination of the P2X1 receptor

To explore the ion permeation pathways of the P2X1 receptor, we utilized single-particle cryo-EM to resolve the structures of the P2X1 receptor at three different calcium ion concentrations (1, 5, and 10 mM). We introduced the full-length, wild-type mouse P2X1 (mP2X1) receptor with a C-terminal Flag tag into an insect baculovirus expression system. This mouse receptor shares 97% sequence similarity with its human ortholog and is readily cloned from the cDNA library. Lauryl maltose neopentyl glycol (LMNG) was used as the detergent to optimize cryo-EM sample quality (Supplementary Fig. S1a, b). The initial cryo-EM data revealed biased particle orientations. We overcame this problem by developing a strategy to enhance the signals from particles with weaker orientations, similar to the previous data processing workflow of multireference-guided 3D classification^27^. This process improved the map quality and overall structural resolution (Supplementary Fig. S1c).

**Fig. 1:**
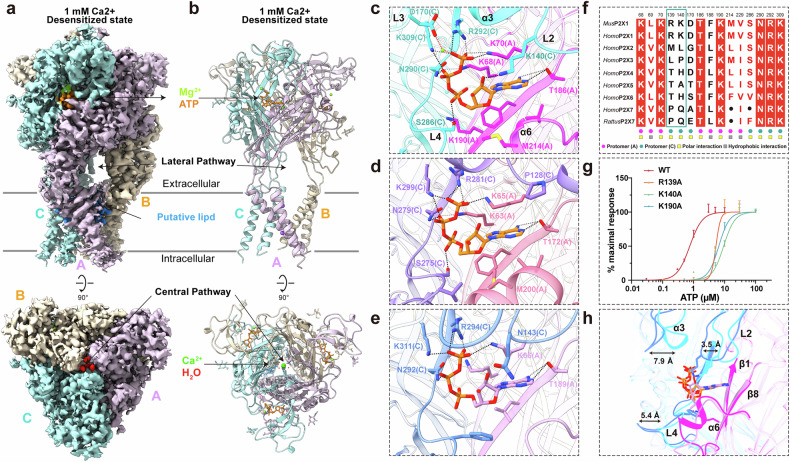
Overall architecture and ATP-binding pocket of the mP2X1 receptor. **a**, **b** Cryo-EM density maps (**a**) and models (**b**) of the mP2X1 receptor in the presence of 1 mM Ca^2+^ viewed from the side (top) and the top (bottom). The three protomers are colored in thistle, blanched almond, and pale turquoise. ATP, Mg^2+^, Ca^2+^, and H_2_O are depicted in dark orange, chartreuse, lime, and red, respectively. **c**–**e** ATP-binding pockets of the mP2X1 receptor (**c**) the human P2X3 receptor (**d**) and the rat P2X7 receptor (**e**) with polar interactions depicted by dashed lines. **f** Sequence alignment of key residues in the ATP-binding pocket of the P2X1 receptor and other P2X receptor family members. Hydrophobic residues and hydrophilic residues are colored gray and yellow, respectively. Additional polar interactions between ATP and the P2X1 receptor are framed in a green box. **g** Concentration‒response curves for ATP activation of the mP2X1 receptors: wild type (red circles, *n* = 3–6), R139A (orange squares, *n* = 3–6), K140A (green triangles, *n* = 3–4), and K190A (blue triangles, *n* = 3–5), normalized to the maximal response. Smooth curves were constructed by fitting the data to the Hill equation, with EC_50_ values and 95% confidence intervals of 0.8288 (0.6257–1.131) µM for the wild type, 5.295 (3.907–7.889) µM for R139A, 10.35 (8.66–12.59) µM for K140A, and 6.736 (4.282–9.991) µM for K190A. **h** Comparison of the ATP-binding caves of the mP2X1 receptor and the rat P2X7 receptor. Protomer A and protomer C of the mP2X1 receptor are colored in magenta and cyan, respectively, and protomer A and protomer C of the rat P2X7 receptor are colored in plum and cornflower blue, respectively.

Remarkably, the EM maps revealed the presence of three clear ATP molecules within the structures even in the absence of exogenous ATP (Fig. 1a, b; Supplementary Fig. S3b). This unexpected result persisted even after the introduction of the ATP-hydrolyzing enzyme apyrase (Supplementary Fig. S2a). Unlike the majority of P2X1 receptor structures, which predominantly exhibit a desensitized state (Supplementary Figs. S1c, d, S2a, b), the structure of the P2X1 receptor bound to the inhibitor BTFA, which was discovered through our structure-based screen as described later, displays a slightly different conformation (Supplementary Fig. S2c). These complexes were characterized by a homotrimer assembly at resolutions from 2.3 Å to 3.2 Å (Supplementary Fig. S2d–h and Table S1).

### Overall architecture of the P2X1 receptor

The trimeric structure of the P2X1 receptor exhibits an architecture that provides new insights into ion permeation and selectivity. Each P2X1 receptor protomer comprises two transmembrane helices (TMs), and three TM2s together form an ion-permeable pathway (Fig. 1a, b). Notably, within each protomer, the TM1 is positioned along the outer edge of the TM2 and displays an approximately 17° counterclockwise rotation relative to it, a feature that may contribute to the regulation of ion permeation. A key finding in the P2X1 receptor structures is the presence of ion-like densities at the top of the upper vestibule (Fig. 1a, b). Consistent with other P2X receptor structures^1,28^, ATP and magnesium ions are bound within the orthosteric pocket formed by the extracellular domains of two adjacent protomers, and *N*-linked glycosylation sites are also observed (Fig. 1a, b; Supplementary Fig. S3). The cytoplasmic domain, including both the N-terminal and C-terminal regions, remains largely unresolved.

### Basis for the high ATP sensitivity of the P2X1 receptor

Despite the shared structural framework across all P2X receptors, the P2X1 receptor displays markedly greater ATP sensitivity and rapid desensitization, distinguishing it from other subtypes, such as the P2X2 receptor and P2X4 receptor, and even more so from the P2X7 receptor, which requires millimolar amounts of ATP for activation and does not become desensitized. Understanding the structural basis of these unique functional features of the P2X1 receptor is therefore essential. Through structural analysis of the ATP-binding pocket in the mP2X1 receptor, we identified several distinctive features that collectively contribute to its high ATP sensitivity.

A key discovery is the presence of additional polar interactions between ATP and residues R139 and K140 of the P2X1 receptor. These residues interact with the ATP molecule and the M214–C217 region, either directly or via water molecules (Fig. 1c; Supplementary Fig. S4a). These interactions are unique to the P2X1 receptor and are not observed in the other P2X receptor subtypes. Mutation of R139 or K140 led to a substantial decrease in ATP-evoked potency, reducing it by 6-fold and 13-fold, respectively, compared with that of the wild type (Fig. 1g; Supplementary Fig. S4b). These findings highlight the critical role of these residues in increasing the ATP sensitivity of the P2X1 receptor.

Furthermore, compared with those of the P2X3 and P2X7 receptors, the structural components of the ATP-binding cave in the P2X1 receptor, including the β1 sheet, the β8 sheet, and the ribose moiety of ATP, are positioned closer together (Fig. 1h). This proximity creates a shorter and more direct pathway for an ATP molecule to enter the interior of the cave from the exterior, facilitating more frequent and stable interactions between the ATP molecule and the receptor. This structural arrangement allows the ATP molecule to bind more readily and become trapped within the P2X1 receptor, contributing to its high sensitivity and rapid desensitization.

In addition to these unique features, the P2X1 receptor shares some conserved polar and hydrophobic interactions with the other P2X receptor family members (Fig. 1c–f). However, the P2X1 receptor and the P2X3 receptor form additional polar interactions with ATP via D170 (D158 in the P2X3 receptor) and S286 (S275 in the P2X3 receptor), which are absent in the P2X7 receptor. These additional interactions further increase ATP sensitivity relative to that of the P2X7 receptor.

### Calcium ion permeation preference of the P2X1 receptor

**Fig. 2:**
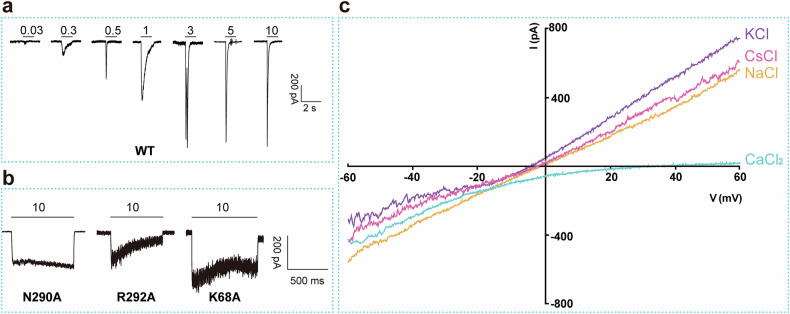
Electrophysiological characterization of the mP2X1 receptor. **a** ATP-induced P2X1 receptor currents undergo rapid desensitization. **b** Key residues located at the orthosteric binding pocket of the P2X1 receptor involved in the slow desensitization evoked by 10 μM ATP. **c** Voltage ramps (–60 to +60 mV in 500 ms) were applied to generate current–voltage curves of the unsusceptible desensitizing P2X1 receptor mutant K68A in bath solutions with the indicated cationic compositions. Recording electrodes were filled with NaCl. Similar results were obtained with the KCl- or CsCl-filled electrodes. Replacement of extracellular NaCl (140 mM) with equimolar KCl or CsCl did not significantly shift the reversal potential (*E*_rev_ = –1.9 ± 1.0 mV, *n* = 3; –1.9 ± 0.9 mV, *n* = 10; –4.1 ± 0.4 mV, *n* = 9, respectively; P_K_/P_Na_ = 0.97; P_Cs_/P_Na_ = 0.85). Replacement of extracellular NaCl with isotonic (112 mM) CaCl_2_ shifted *E*_rev_ to 25.3 ± 2.2 mV (*n* = 11; P_Ca_/P_Na_ = 10.40).

To assess the selectivity of the P2X1 receptor for calcium ions (Ca^2+^) over sodium ions (Na^+^) and other cations, we performed current‒voltage relationship experiments using a novel approach leveraging the slow desensitization of specific orthosteric pocket mutants. We identified three mutants that exhibit slow desensitization: N290A, R292A, and K68A (Fig. 2a, b). Focusing on the K68A mutation, we observed a dramatic decrease in ATP potency, resulting in an outwardly rectifying current‒voltage profile and a reversal potential near 0 mV (*E*_rev_ = 0.5 ± 0.9 mV, *n* = 13) (Fig. 2c). This finding is significant because it allows for a more accurate assessment of ion permeation properties without the confounding effects of rapid desensitization.

We conducted ion substitution experiments to elucidate the contributions of various cations to ATP-evoked currents in cells overexpressing the P2X1-K68A mutant. Our results revealed that while the P2X1 receptor does not exhibit selectivity among monovalent cations, it exhibits a significantly higher permeability to calcium, with a calcium-to-sodium permeability ratio (P_Ca_/P_Na_) of 10.6 (Fig. 2c). This value is consistent with previously reported data^16^, validating our experimental approach and confirming the calcium ion permeation preference of the P2X1 receptor.

### Central ion pathway in the P2X1 receptor

Cation permeation in P2X-ENaC superfamily channels is hypothesized to occur via the lateral fenestrations of the central vestibule^29–31^, followed by traversal across the membrane into the cytoplasm. The small size of the threefold axis orifice at the top of the central vestibule restricts direct ion flow through the central pathway (Fig. 1a, b; Supplementary Fig. S5a). Despite extensive studies^32^, how P2X receptors confer cation selectivity, and, in particular, the calcium selectivity of P2X1, remain incompletely understood. To investigate the mechanism of calcium selectivity of the P2X1 receptor, we used different concentrations of calcium ions to identify calcium binding and selectivity sites.

**Fig. 3:**
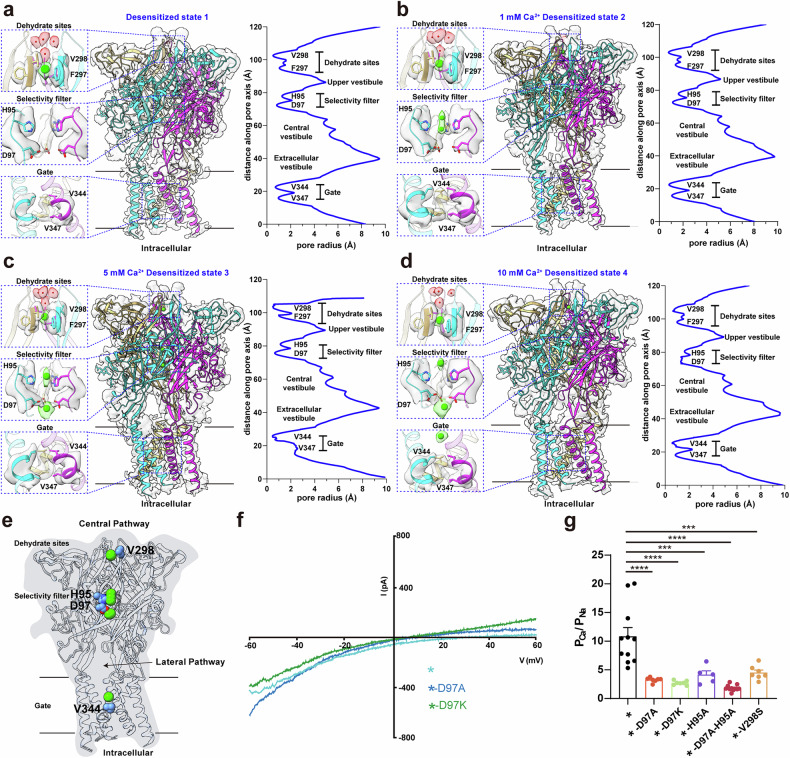
Central calcium ion permeation pathway of the mP2X1 receptor. **a**–**d** Structural model superimposition within the density map of the mP2X1 receptor in its desensitized state 1 (**a**) 1 mM Ca^2+^ desensitized state 2 (**b**) 5 mM Ca^2+^ desensitized state 3 (**c**) and 10 mM Ca^2+^ desensitized state 4 (**d**). The left panel displays, from top to bottom, the dehydration site, the selectivity site, the local gating model, and the corresponding density map. The plot on the right illustrates the radius of the central pathway, extending from the extracellular tip to the cytoplasmic side. **e** Calcium ion superposition maps appearing in the central pathway of the P2X1 receptor at different calcium ion concentrations. **f** Mutation of residue D97 to alanine (denoted *-D97A) or lysine (denoted *-D97K) caused a significant rightward shift in the reversal potential (from 25.3 ± 2.2 mV (*n* = 11) to 9.0 ± 0.8 mV (*n* = 7) and 6.1 ± 0.8 (*n* = 7), respectively). *represents the slow desensitization mutant K68A. **g** Mutation of the dehydration site and selectivity filter reduced calcium selectivity along the central pathway of the P2X1 receptor in response to 10 μM ATP. Data are presented as the means ± SEM. ***P < 0.001, ****P < 0.0001, two-way ANOVA with post hoc Tukey’s test (*n* = 5–10 cells).

High-resolution cryo-EM maps obtained for different calcium ion concentrations allowed us to model the central pathway along the central axis of the P2X1 receptor. We traced the pathway continuously, including the density at the apex of the extracellular domain, the upper vestibule, the central vestibule, and the gate (Fig. 3a–d). In addition to the well-defined protein density, we also observed ion-like densities along the central pathway.

A density corresponding to a hydrated ion was identified at the midpoint of the three V298 main chain carbonyl oxygens (Fig. 3a–d), resembling the hydrated sodium ion densities observed in the TRPA1 channel^33^. In the P2X1 receptor structure obtained in the presence of 1 mM calcium ions, two consecutive ion densities were observed at the threefold axis near the imidazole group of H95. In contrast, no such densities were observed in desensitized state 1, which was obtained without the addition of exogenous ATP or Ca^2+^; however, electron densities corresponding to ATP molecules were still observed in the structure (Fig. 3a, b). As the calcium ion concentration increased to 5 mM, increased ion-like densities were detected beneath the anionic ring formed by D97 at the top of the central vestibule, leading to a more tightly packed ring structure than that in the calcium-free state (Fig. 3c). At 10 mM calcium, an additional ion density appeared below a putative lipid and above the gate, influencing the radii of V344 and V347 (Fig. 3d). This density was absent in desensitized state 1 and at lower calcium concentrations (1 mM and 5 mM). On the basis of these observations, we proposed that these additional densities correspond to calcium ions, whose presence depends on the calcium concentration (Fig. 3a–e).

Functional mutagenesis further supported the role of specific residues in calcium ion selectivity (Fig. 3f, g). The V298S mutation reduces calcium ion selectivity, as substituting valine with serine introduces a more electronegative group compared to the original isopropyl side chain, thereby reducing calcium ion permeation. Similarly, the H95A mutation disrupts divalent cation coordination with the imidazole group, weakening calcium ion selectivity. Mutation of D97 to alanine or lysine significantly reduces calcium ion permeability, with the double mutation D97A/H95A causing an even greater loss of selectivity.

By analyzing the distribution of the proposed calcium ion densities from the upper vestibule to the extracellular vestibule under varying calcium concentrations and correlating these findings with data from mutations affecting calcium ion selectivity (Fig. 3e), we proposed the central pathway as one of the routes for calcium ion permeation in the P2X1 receptor. The presence of water density near the apex of the central pathway and the impairment of calcium ion permeability upon V289 mutation suggest that this region functions as a dehydration zone (Fig. 3a–e). Additionally, aspartic acid residues are well-known contributors to selectivity filters in ion channels such as CaV channels^34^, TRP channels^3^, and nicotinic acetylcholine receptors^35^. In the P2X1 receptor, H95 and D97, located at the top of the central vestibule, play critical roles in calcium ion selectivity (Fig. 3a–e), as demonstrated by the profound effects observed upon their mutation. Molecular dynamics simulations revealed that calcium ions can move from the upper vestibule past the selectivity site (H95–D97) into the central and extracellular vestibules (Supplementary Fig. S6e, f). Importantly, these calcium ions do not leak out of the extracellular vestibule region (Supplementary Videos S1–S3). We therefore designate D97 as the calcium ion selectivity filter of the central pathway. These findings highlight a complex regulatory mechanism governing calcium ion dehydration, selectivity, and permeation in the P2X1 receptor.

During cryo-EM data processing, discrepancies in the transmembrane domain densities were observed among different 3D classes of the P2X1 receptor in the presence of 10 mM calcium (Fig. 3d; Supplementary Fig. S6a, b). After the maps with preferred orientations and incomplete transmembrane domain densities were excluded, three distinct maps remained. In addition to differences in the distribution of ion-like densities in the extracellular vestibule, slight rotational shifts were observed in the other models (Supplementary Fig. S6c, d). However, these microdisturbances were minor, and no notable changes were observed in the cytoplasmic region. Therefore, these states cannot be defined as new conformational states and were collectively assigned to the desensitized state.

### BTFA inhibits cation permeation at the central vestibule of the P2X1 receptor

The majority of antagonists of the P2X receptor family are ATP analogs^1,17,36–38^. The conserved consensus amino acids in the ATP pocket contribute to the reduced selectivity of these orthosteric antagonists for P2X receptor subtypes compared with allosteric antagonists. On the basis of our discovery of a central permeation pathway and calcium ion preference sites, we conducted small-scale virtual docking targeting the pocket at the top of the central vestibule to identify allosteric inhibitors. Screening a library of 4000 compounds filtered for interactions with D97 led to the identification of a small molecule, BTFA (a segment of IMD-0354 containing the trifluoromethyl group^39^), with a promising docking score (–4.838 kcal/mol). BTFA inhibited the mixed-cation current of the mP2X1 receptor under extracellular recording conditions (147 mM NaCl, 2 mM KCl, 2 mM CaCl_2_, 1 mM MgCl_2_, 10 mM HEPES (pH 7.3), and 13 mM glucose), yielding an IC_50_ of 4.301 μM (Supplementary Fig. S7a, b). Consistently, calcium imaging in HEK293 cells overexpressing mP2X1 using the fluorescent dye Fluo-4 AM confirmed that BTFA also suppresses ATP-induced Ca^2+^ influx (Supplementary Fig. S7c).

**Fig. 4:**
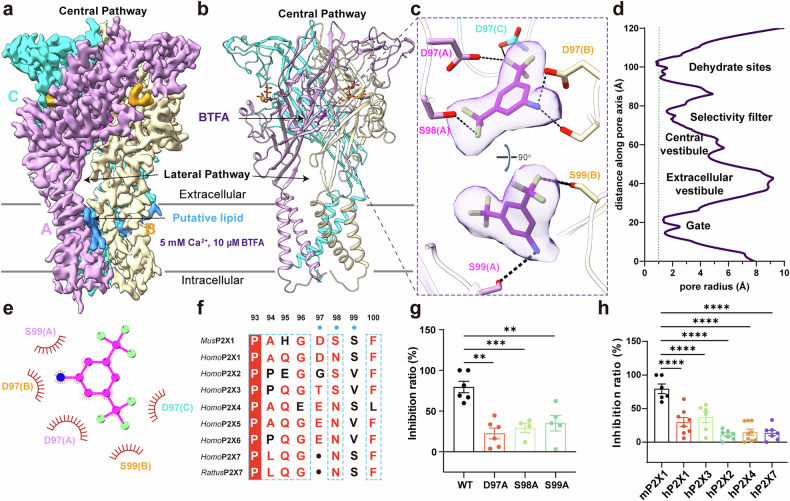
BTFA bound at the top of the central vestibule of the mP2X1 receptor can inhibit the permeation of cations. **a**, **b** Cryo-EM density map (**a**) and model (**b**) of the mP2X1 receptor in the presence of 5 mM Ca^2+^ and 10 μM BTFA. BTFA is colored in Rebecca purple. **c** Binding pocket of BTFA to the mP2X1 receptor viewed from the side (top) and the top (bottom); the density of BTFA is shown in semitransparent gray. **d** The radius of the central pathway of the mP2X1 receptor in the presence of 10 μM BTFA. **e** Binding pocket of BTFA and the mP2X1 receptor depicted by LigPlus. **f** Sequence alignment of key residues in the BTFA-binding pocket of the mP2X1 receptor and human P2X receptor family members. **g** Effects of BTFA (30 μM) on ATP (10 μM)-evoked currents of the mP2X1 receptor and its mutants D97A, S98A, and S99A (means ± SEM, *n* = 4–6). Significance was assessed using one-way ANOVA with Tukey’s multiple comparison test. All the quantitative data are presented as the means ± SEM. **P < 0.01, ***P < 0.001. **h** Representative effects of 30 μM BTFA on human P2X receptor subtypes (*n* ≥ 6). Significance was assessed using one-way ANOVA with Tukey’s multiple comparison test. All the quantitative data are presented as the means ± SEM. ****P < 0.0001.

To validate this predicted binding mode, we determined the cryo-EM structure of the P2X1–BTFA complex. The density of the BTFA-bound P2X1 receptor structure revealed that BTFA binds to the top of the central vestibule, as expected (Fig. 4a, b; Supplementary Fig. S8a–c). The binding site of the mP2X1 receptor for BTFA consists of polar residues within the central vestibule, notably D97 from all three protomers and S99 from protomer A and S98 from protomer B (Fig. 4a–c, e). These residues engage in polar interactions with the trifluoromethyl or phenylamine groups of BTFA, stabilizing the formation of the binding pocket (Fig. 4c, e). Single point mutations of these residues (D97A, S98A, and S99A) significantly reduced the sensitivity of the mP2X1 receptor to BTFA, confirming the functionality of this binding site (Fig. 4g).

Having identified the BTFA-binding site, we next examined how this interaction alters receptor conformation and channel gating. The cryo-EM map enabled side chain assignment and allowed modeling of residues V24–G30 in the N-terminal region (Supplementary Fig. S8a–d). Together with the C-terminal segment, the N-terminus contributes to the formation of the cytoplasmic ring, a structural hallmark of P2X receptors in the open state^1,40,41^. In contrast to BX430 and BAY-1797^42^, which act by preventing activation of the P2X4 receptor, BTFA binding results in a concerted rotation of the two TMs (TM1 and TM2) in each protomer, leading to widening of the channel gate without achieving an open conformation (Supplementary Fig. S8e). Moreover, BTFA does not induce closure of the P2X1 channel, unlike NF449^37,38^, which triggers extracellular conformational rearrangements and restores the transmembrane domain to a closed state. Thus, this structure may represent an intermediate state, in which parts of the cytoplasmic region become visible, yet the receptor has not adopted a fully activated state and is different from the desensitized state. (Fig. 4d; Supplementary Fig. S8e). Therefore, we proposed that BTFA inhibits ion permeation by occupying the central vestibule at the top of the central pathway.

To evaluate the selectivity of this novel inhibitor against closely related receptors, we examined the effects of BTFA on other homologous human P2X subtypes (Fig. 4h; Supplementary Fig. S7d). BTFA had virtually no effect on the human P2X2 and human P2X7 receptors, where the critical residue D97 of the P2X1 receptor is either absent or mutated. BTFA also had minimal inhibitory effects on the human P2X3 receptor, attributed to the lack of polar interactions with S99, which corresponds to V89 in the P2X3 receptor. In the P2X4 receptor, the longer side chains of E97 and N98 created steric hindrance that prevented BTFA binding. The inhibitory effect of 10 µM BTFA on the human P2X1 receptor was approximately half that observed for the mP2X1 receptor at the same concentration. This difference is attributed to the substitution of N98 in human P2X1 with S98 in mP2X1, which alters the binding interactions. These findings suggest that the BTFA-binding site, owing to its low sequence conservation, is a promising target for the development of selective antagonists of P2X receptors.

## Discussion

A distinctive feature of the P2X receptor family is the presence of three lateral portals within the extracellular central vestibule. While it is widely accepted that cations enter the P2X3 receptor^1^ and P2X4 receptor^43^ through lateral fenestrations before they permeate through the membrane into the cytoplasm, previous studies^32,43^ have suggested that the calcium selectivity of P2X receptors is primarily determined by acidic residues located near the extracellular ends of the TMs and around the lateral fenestrations. In the P2X4 receptor^43,44^, two adjacent acidic residues, D59 and D61, positioned near the lateral fenestrations, facilitate local cation accumulation. In contrast, the corresponding residues in the P2X1 receptor, T57 and S59, are neutral. Consistently, similar ion densities were observed at the top of the central vestibule for both the P2X4 receptor and the P2X1 receptor. Notably, in the P2X1 receptor, additional ion densities were detected within the upper region of the central vestibule, and their distribution along the central axis varied with the extracellular calcium concentration. Furthermore, D97, located at the top of the central vestibule, plays a critical role in calcium selectivity rather than acting primarily as a cation concentration site, as reported for the P2X4 receptor^43^. Taken together, these observations support the notion that a central pathway spanning the entire extracellular domain constitutes a cation permeation route in the P2X1 receptor. This finding complements previous models of P2X receptor permeation pathways and ion selectivity. Moreover, our results suggest that the ion selectivity of P2X receptors may arise from the coordinated contributions of multiple distributed structural elements, whose individual mechanisms and relative contributions to ion selectivity remain to be elucidated.

Notably, BTFA was identified as an inhibitor that binds to the top of the central vestibule and inhibits cation flux in a dose-dependent manner. This binding site overlaps with the Gd^3+^-binding site in the P2X4 receptor, where Gd^3+^ is known to block cation permeation. Unlike conventional channel blockers^45–47^ that directly obstruct the transmembrane pore, BTFA binds to the extracellular domain without inducing detectable conformational changes in the extracellular region. However, the inhibition mechanism of BTFA remains to be determined. Moreover, even at high concentrations, BTFA does not completely abolish P2X1 currents, indicating that metal ions can still permeate lateral fenestrations. Consequently, the relative contributions of the central pathway and lateral fenestrations to overall ion permeation and ion selectivity warrant further investigation.

The P2X1 receptor demonstrates a preference for cholesterol-rich lipid rafts in arteries and platelets^48^. Depletion of cholesterol and phosphatidylinositol-4,5-bisphosphate significantly reduces P2X1 receptor current amplitude^49^. In our study, lipid-like densities were observed at each protomer–protomer interface in all P2X1 receptor structures. These densities are located on the extracellular side of the transmembrane domain and do not directly block the canonical lateral gate formed by V344 and V347. Instead, they likely form a separate structural feature resembling a gate (Supplementary Fig. S5d). However, owing to limited local map quality and the lack of direct characterization, the identities of these lipid-like densities remain unclear.

Our comparative structural analysis further revealed that the P2X1 receptor shares functional features with other ligand-gated ion channels (Supplementary Fig. S5e, f), such as 5-HT_3_R^50^, nAChR^51^, and chemotactile receptors^52^. In addition to the central pathway, the ATP-binding site is spatially close to the ligand-binding sites of these channels, and the calcium selectivity sites display similar spatial features. Together, our findings support the hypothesis that, beyond the established lateral fenestrations, the central vestibule spanning the extracellular domain also contributes to cation permeation in the P2X1 receptor.

## Materials and methods

### Expression and purification of mP2X1

The mP2X1 gene was cloned from cDNA extracted from the olfactory cortex of a mouse. After sequencing, the sequence was confirmed to be consistent with that of wild-type mouse P2X1 (*P2RX1*; UniProt: P51576). The gene was then inserted into the pFastBac vector (Thermo Fisher Scientific) using the ClonExpress II One-Step Cloning Kit (Vazyme Biotech). His tag and 1× Flag tag were positioned at the C-terminus of the proteins and connected by an HRV-3C protease recognition site. mP2X1 was expressed in SF9 (*Spodoptera frugiperda*) cells using the Bac-to-Bac expression system (Gibco). SF9 cells were cultured in ESF921 medium (expression system) at 27 °C and 120 rpm until they reached a density of approximately 2.5 million cells/mL. Subsequently, 20 mL of P1 virus was added to 1 L of SF9 cells. After 72 h post-infection, the cells were harvested by centrifugation at 2000× *g* for 15 min and stored at –80 °C.

Cell disruption was carried out using a Dounce tissue grinder (Merck Millipore) in buffer solution (50 mM HEPES (pH 7.3), 150 mM NaCl, and 10% glycerol) supplemented with a mixture of the following protease inhibitors: 10 µg/mL aprotinin, 4 µg/mL leupeptin, and 3 µg/mL pepstatin A (all from Yeasen Biotechnology (Shanghai) Co., Ltd). A final concentration of 1% (w/v) *n*-dodecyl-d-maltoside (DDM; Anatrace) containing 0.1% (w/v) cholesteryl hemisuccinate (CHS; Anatrace) was added to extract the membrane components for 3 h at 4 °C. Insoluble components were removed by centrifugation (20,000× *g*, 1 h, 4 °C). The detergent-soluble fraction was then incubated for 2 h at 4 °C with anti-DYKDDDDK G1 Affinity Resin (GenScript). The protein was subsequently eluted using 0.2 mg/mL DYKDDDDK peptide (GenScript) and purified by size-exclusion chromatography (SEC) on a Superose6 Increase 10/300 GL column (GE HealthCare) equilibrated with SEC buffer (20 mM HEPES (pH 7.0), 150 mM NaCl, and 0.002% LMNG). The fractions of the protein were collected and concentrated to 4.0 mg/mL using a 100 kDa MWCO Amicon centrifugal filter (Merck Millipore). To determine the structure of P2X1 in the presence of various concentrations of calcium, buffers with different calcium concentrations (1 mM, 5 mM, and 10 mM) were used. To determine the structure of the P2X1–BTFA complex, 10 μM BTFA was added throughout the entire purification process.

### Grid preparation and data acquisition

To determine the single-particle cryo-EM structure of mP2X1, a 2.1 μL aliquot of purified protein solution at a concentration of 4.0 mg/mL was applied to glow-discharged holey carbon grids (Quantifoil R1.2/1.3 Au 300 mesh, Coolglow, SuPro). Excess liquid was removed using a Vitrobot Mark IV (Thermo Fisher Scientific) at 8 °C and 100% humidity by blotting grids for 4.5 s with a blotting force of 1. The grids were subsequently flash-frozen in liquid ethane.

The frozen grids were transferred to a 300 kV Titan Krios G4 microscope (FEI) equipped with a cold field emission gun (E-CFEG) and a Falcon 4i direct electron detector at a pixel size of 0.73 Å or a Gatan K3 Summit direct electron detector at a pixel size of 0.824 Å. Raw micrographs were collected using EPU (v2.13) at the Shanghai Advanced Center for Electron Microscopy. All the datasets were recorded with a total dose of 50 electrons per square angstrom (e^–^/Å^2^) for 36 frames. Raw images were aligned using the motion correction program MotionCor2.

### Data processing

Micrographs that underwent motion correction were transferred to cryoSPARC (v3.2). Patch CTF was used for contrast transfer function (CTF) estimation. The micrographs with a CTF resolution poorer than 4 Å were discarded. The data processing workflow referenced previous publications, with the addition of a strategy to enhance weak orientations. We identified weakly oriented images on the basis of the 2D classification results and created a separate dataset consisting of weakly oriented images. The best category from this dataset was selected and incorporated into each iteration of the multireference refinement process, and duplicate particles were removed during each cycle. This process continued iteratively until a noticeable increase in the dominant orientation was achieved.

For the P2X1-desensitized state 1 dataset, 2900 dose-weighted micrographs were imported into cryoSPARC; after the CTF was estimated, 1,324,704 particles were extracted using blob picker. Following three rounds of 2D classification, particles exhibiting clear second structural features were selected for ab initio reconstruction and local refinement. The best model was chosen as a good reference. After multiple rounds of multireference 3D classification, a 3.2 Å map was reconstructed from 119,704 particles. However, this map displayed a preferential orientation. To improve the map’s overall quality, we attempted an iterative approach to enhance reconstruction from weak orientations, resulting in a 3.5 Å map with an improved preferred orientation. Using this map as a template, we repicked the particles and performed multiple rounds of multireference 3D classification and refinement, yielding a 2.45 Å map. Further resolution enhancement was achieved by applying C3 symmetry, resulting in an overall map resolution of 2.31 Å.

For the other datasets, we followed the same strategy. However, unlike the P2X1-desensitized state 1 dataset, the datasets of P2X1 with 5 mM calcium ions, P2X1 with 10 mM calcium ions, and P2X1 with 10 μM BTFA did not utilize C3 symmetry for resolution enhancement.

### Model building and refinement

The initial structural model of mP2X1 was generated using AlphaFold2. The model matched with the P2X3 model was subsequently fitted onto the density map using UCSF ChimeraX. Through iterative manual adjustments and reconstruction in COOT, the model was refined to closely match the density map. Ligand restraint files were generated using PHENIX1.17_elbow. Finally, the refined models were further optimized against the corresponding maps using phenix.real_space_refine.

### Docking small molecules into P2XR1

Using the cryo-EM structure of the P2X1 receptor in the desensitized state as the docking template, we first defined the central vestibule pocket by mapping its geometric boundaries and evaluating local physicochemical features, including the pocket volume, polarity distribution, and electrostatic potential. Residues forming the vestibule, particularly D97, were examined for side chain accessibility and the potential to mediate ligand interactions.

A small library of ~4000 small molecules (molecular weight < 500 g/mol) from TopScience was constructed. The library was curated to enrich chemical scaffolds predicted to interact with acidic residues, thereby increasing the likelihood of engaging D97. All compounds were prepared using standard protonation and conformer generation protocols prior to docking. Molecular docking was performed using DOCK 3.6, which evaluates ligand poses through physics-based scoring integrating van der Waals complementarity, electrostatics, and ligand desolvation penalties. Candidate poses were ranked according to predicted binding energy and the presence of favorable interactions with conserved vestibule-lining residues. Only one compound, BTFA, achieved a docking score exceeding the preset threshold of –3.0 kcal/mol, with a score of –4.838 kcal/mol. Docking analysis predicted that BTFA engages the central vestibule via hydrogen bonding and hydrophobic contacts involving D97 and adjacent residues, suggesting a plausible mode of binding within this pocket. BTFA was subsequently selected for functional validation using electrophysiological assays on the P2X1 receptor.

### Whole-cell patch-clamp electrophysiological recordings

Wild-type and mutant mP2X1, which were subcloned and inserted into the pCDNA3.1 vector, were labeled with mCherry as an indicator for subsequent electrophysiological recordings. Whole-cell patch-clamp recordings were performed on HEK293 cells at room temperature (25 ± 2 °C) 24–48 h after transfection with Lipofectamine 3000 (Thermo Fisher Scientific, USA) using a Multiclamp 700B amplifier (Axon Instruments, USA) and a Digitata 1440 A digitizer (Axon Instruments, USA). Currents were sampled at 10 kHz and low-pass filtered at 2 kHz. Patch electrodes were pulled from borosilicate glass capillaries (World Precision Instruments) and had resistances of 1.5–4 MΩ when filled with intracellular solution (145 mM NaCl, 10 mM EGTA, 10 mM glucose, and 10 mM HEPES (adjusted to pH 7.3 with NaOH)). The extracellular solution was composed of 147 mM NaCl, 2 mM KCl, 2 mM CaCl_2_, 1 mM MgCl_2_, 10 mM HEPES, and 13 mM glucose (adjusted to pH 7.3 with NaOH). ATP solutions were preprepared in batches no more than 2 h prior to use and applied through an RSC-200 Rapid Solution Changer with a 9-tube head (BioLogic, France), in which the cell was continuously superfused by means of a close delivery system consisting of pipettes (tip diameter 500 μm) placed ~300 μm away. This allowed switching to a solution containing drug or ATP in 10 ms.

After the whole-cell configuration in the standard solution was obtained, the extracellular solution was changed to a solution containing 140 mM NaCl (or KCl or CsCl), 10 mM glucose, and 10 mM HEPES (adjusted to pH 7.4 with NaOH, KOH, or CsOH, respectively), and the intracellular solution was correspondingly changed to a solution containing 140 mM NaCl (or KCl or CsCl), 5 mM EGTA, and 10 mM HEPES (adjusted to pH 7.4 with NaOH, KOH, or CsOH, respectively) for the monovalent cation preference experiments. The extracellular solution for divalent cation permeability experiments was composed of 110 mM MgCl_2_ (or CaCl_2_), 2 mM Mg(OH)_2_ (or Ca(OH)_2_), 10 mM glucose, and 10 mM HEPES (adjusted to pH 7.4 with HCl). The reversal potential was measured by recording the current‒voltage data during a 1 s voltage step from –60 mV to 60 mV with 10 µM ATP applied.

The relative permeabilities were calculated as follows:

$${P}_{X}/{P}_{{Na}}=\exp (\Delta {\varPsi }_{{rev}}F/{RT})$$

$${P}_{Y}/{P}_{{Na}}={[{{Na}}^{+}]}_{i}\exp ({\Psi }_{{rev}}F/{RT})(1+\exp ({\Psi }_{{rev}}F/{RT}))/4{[{Y}^{2+}]}_{o}$$

where P_Na_, P_X_, and P_Y_ are the permeabilities to Na^+^, monovalent cations, and divalent cations, respectively; Ψ_rev_ is the measured reversal potential; F is Faraday’s constant; R is the universal gas constant; and T is the absolute temperature. The bracketed terms are ion activities, where it is assumed that the activity coefficients are 0.75 for monovalent ions and 0.25 for divalent ions. This approach allowed us to quantify the ion selectivity of the P2X1 receptor with high precision.

### Calcium assay

HEK293 cells were transfected with mCherry-P2XR1 and seeded at a density of 4 × 10^4^ cells per well into 96-well culture plates and incubated for 24 h at 37 °C in 5% CO_2_. The cells (*n* = 6 samples) were then incubated with 2 μmol/L Fluo-4 AM in HBSS (5.4 mmol/L KCl, 0.3 mmol/L Na_2_HPO_4_, 0.4 mmol/L KH_2_PO_4_, 4.2 mmol/L NaHCO_3_, 1.3 mmol/L CaCl_2_, 0.5 mmol/L MgCl_2_, 0.6 mmol/L MgSO_4_, 137 mmol/L NaCl, 5.6 mmol/L d-glucose and 250 μmol/L sulfinpyrazone; pH 7.4) at 37 °C for 40 min. After thorough washing, 50 μL of HBSS was added. After incubation with various concentrations of BTFA and vehicle at room temperature for 10 min, 10 μM ATP was dispensed into the wells using a FlexStation III microplate reader (Molecular Devices), and the change in intracellular calcium concentration was determined by recording the fluorescence intensity at an excitation wavelength of 485 nm and an emission wavelength of 525 nm.

### Molecular dynamics simulations

The initial protein models were constructed on the basis of the cryo-EM structures of P2X1 in four distinct conformational states: P2X1-desensitized state 1 (PDB: 8ZT2), 1 mM Ca^2+^-bound state 2 (PDB: 8ZT5), 5 mM Ca^2+^-bound state 3 (PDB: 8ZT8), and 10 mM Ca^2+^-bound state 4 (PDB: 8ZTA). These models were inserted into a palmitoyl-2-oleoyl-sn-glycero-3-phosphocholine (POPC) membrane using CHARMM-GUI Membrane Builder, with a 22.5 Å water layer added and neutralized using CaCl_2_ at 10 mM, 1 mM, 5 mM, and 10 mM for the respective states.

All-atom molecular dynamics simulations were performed using Amber22, starting with 5000 cycles of energy minimization and applying constraints to the backbone, side chain atoms, lipid coordinates, and dihedral angles. Following minimization, the system was equilibrated in six steps, with the constraints progressively decreasing. Three independent 500 ns production runs were conducted in the NPT ensemble, where the temperature was maintained at 303.15 K using Langevin dynamics, and pressure coupling was implemented via the semi-isotropic Berendsen method (1 atm). Long-range electrostatic interactions were calculated using the particle mesh Ewald method, whereas short-range electrostatic and van der Waals interactions were treated with a 12 Å cutoff, and gradually switching between 10 Å and 12 Å. The SHAKE algorithm was applied to restrain bonds involving hydrogen atoms, allowing for a 2 fs timestep. The pore’s principal axis was aligned along the *z*-axis to facilitate the application of a uniform electric field along the negative *z*-direction from the extracellular to the intracellular side. The electric field strengths varied up to 0.05 V/Å, corresponding to a transmembrane potential of ~50 mV. Ion permeation was then statistically analyzed under these conditions. Trajectory analysis was conducted using the CPPTRAJ package.

## Supplementary information

Supplementary Information

Supplementary Video S1

Supplementary Video S2

Supplementary Video S3

## Data Availability

All data are available in the main text or the Supplementary Information. The density maps and structure coordinates have been deposited to the Electron Microscopy Data Bank (EMDB) and the Protein Data Bank (PDB) under accession numbers: EMD-60445 and PDB-8ZT2 for P2X1-desensitized state 1; EMD-60447 and PDB-8ZT5 for P2X1-1 mM Ca^2+^; EMD-60453 and PDB-8ZT8 for P2X1-5 mM Ca^2+^; EMD-60457 and PDB-8ZTA for P2X1-10 mM Ca^2+^-desensitized state 4; EMD-60458 and PDB-8ZTD for P2X1-10 mM Ca^2+^-desensitized state 5; EMD-60460 and PDB-8ZTF for P2X1-10 mM Ca^2+^-desensitized state 6; EMD-67120 and PDB-9XQR for P2X1-5 mM Ca^2+^-BTFA.

## Materials availability

Materials are available from the corresponding authors upon reasonable request.
