# Supplementary material for: Structural basis for a central permeation pathway in the P2X1 receptor
Source: Cell Discov. 2026 Jun 4;12:41. doi: 10.1038/s41421-026-00881-w (PMC13237026; doi:10.1038/s41421-026-00881-w)
Supplement: Supplementary file 1 — Supplementary Information [file 41421_2026_881_MOESM1_ESM.pdf]

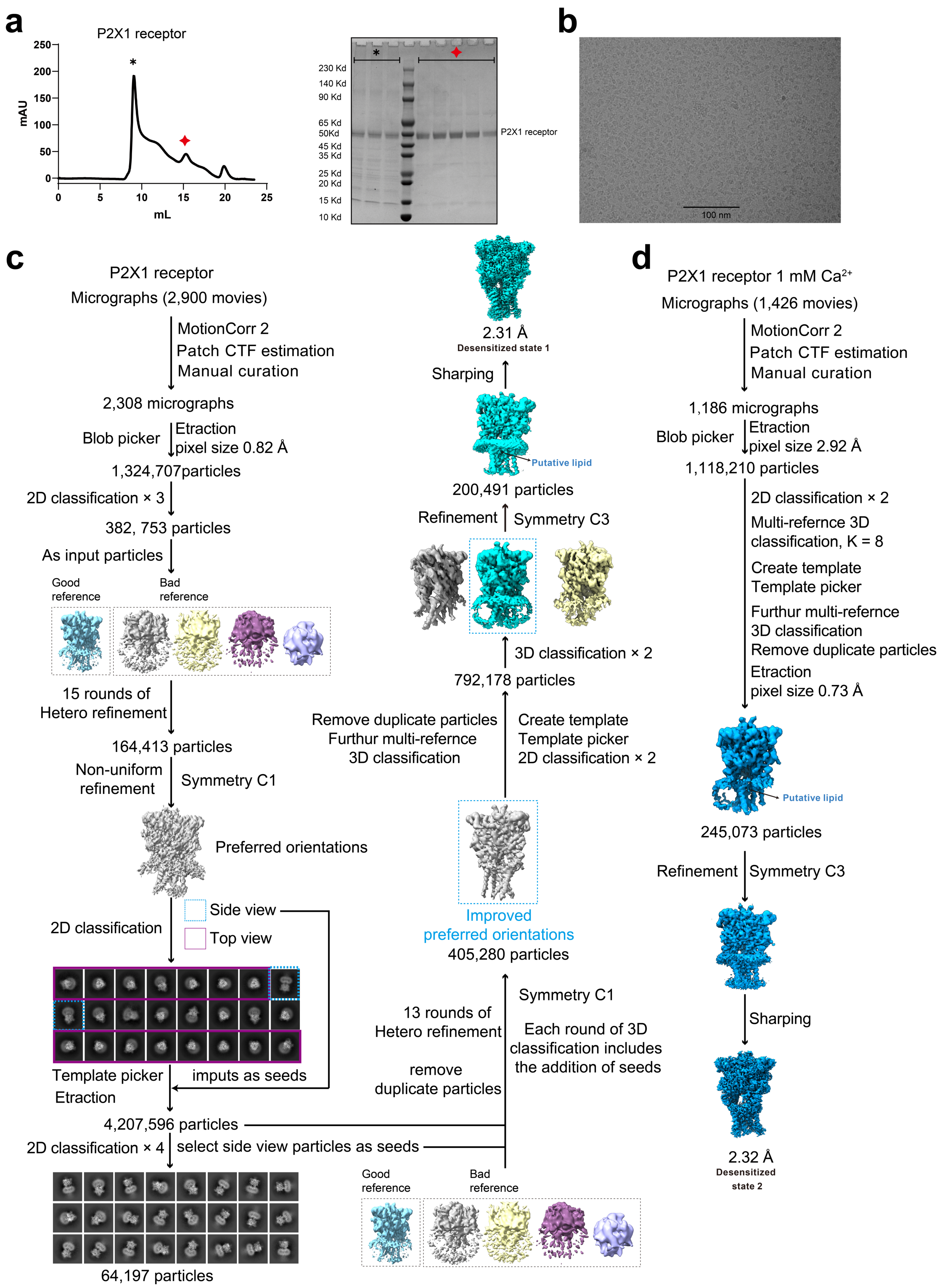

Supplementary Fig. S1 Cryo-electron Microscopy data processing workflows of the mP2X1 receptor. a, The size-exclusion chromatography evaluation (left) and the SDS-PAGE analysis result (right) of the mP2X1 receptor in 0.003% LMNG containing 0.0006% CHS, the fractions indicated by red stars collected to prepare cryo-electron microscopy single-particle samples. **b,** Representative cryo-EM micrographs of mP2X1 receptor. **c,** Data processing workflow of mP2X1 receptor. **d,** Data processing workflow of mP2X1 receptor in the presence of 1 mM Calcium ions.

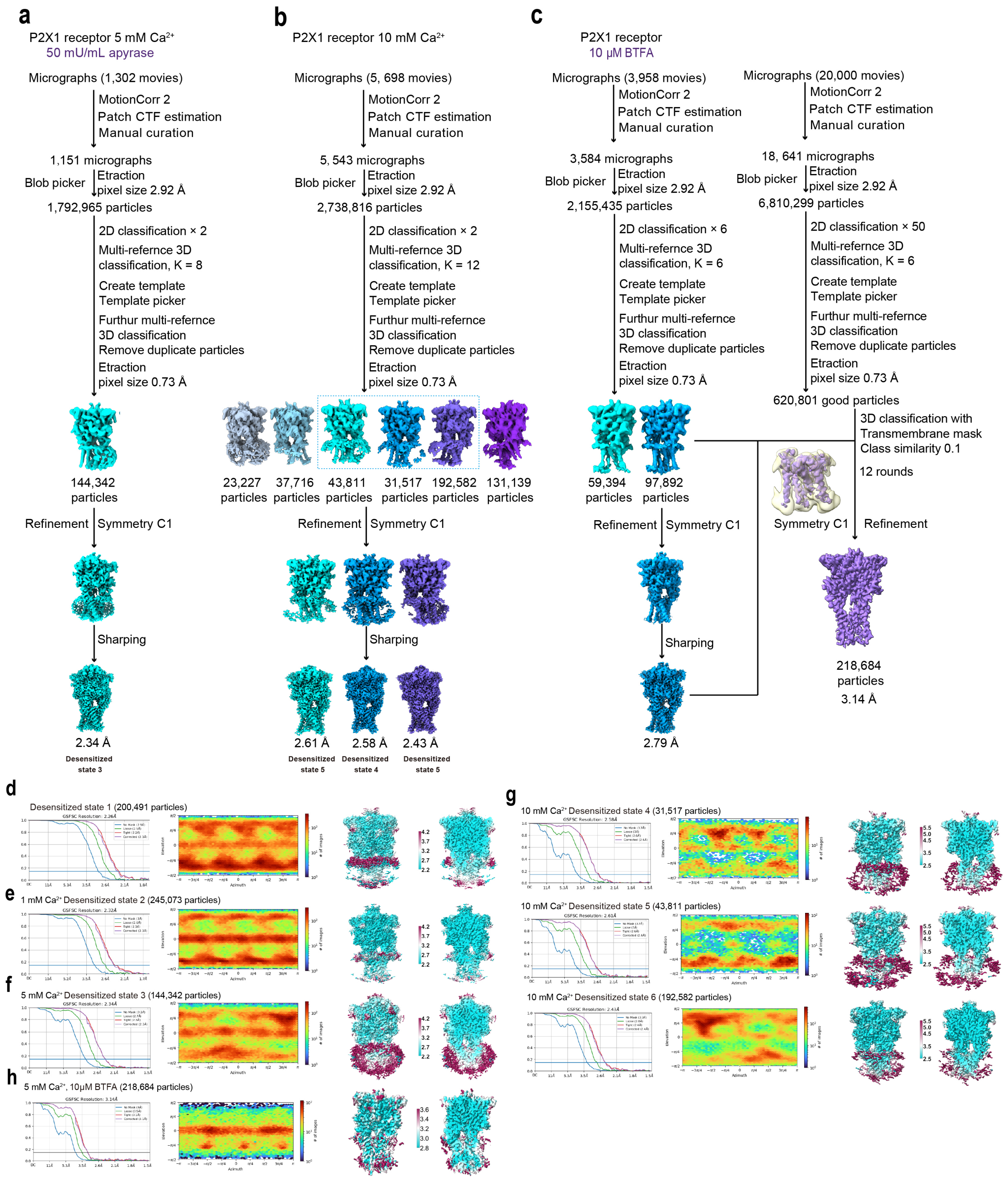

Supplementary Fig. S2 Cryo-electron Microscopy data processing workflows and validations of mP2X1 a, b, c, Data processing workflow of mP2X1 receptor in the presence of 5 mM Calcium ions (**a**), 10 mM Calcium ions (**b**) and 10 μM BTFA (**c**). **d, e, f, g, h,** Gold standard Fourier shell correlation curves for the 3D refinement of the overall structure, angular distributions of the particles used for final reconstitution of the structure and the local resolution of the sharpened maps of mP2X1 receptor in 0 mM Calcium ions (**d**), 1 mM Calcium ions (**e**), 5 mM Calcium ions (**f**), 10 mM Calcium ions (**g**) and 10 μM BTFA (**h**).

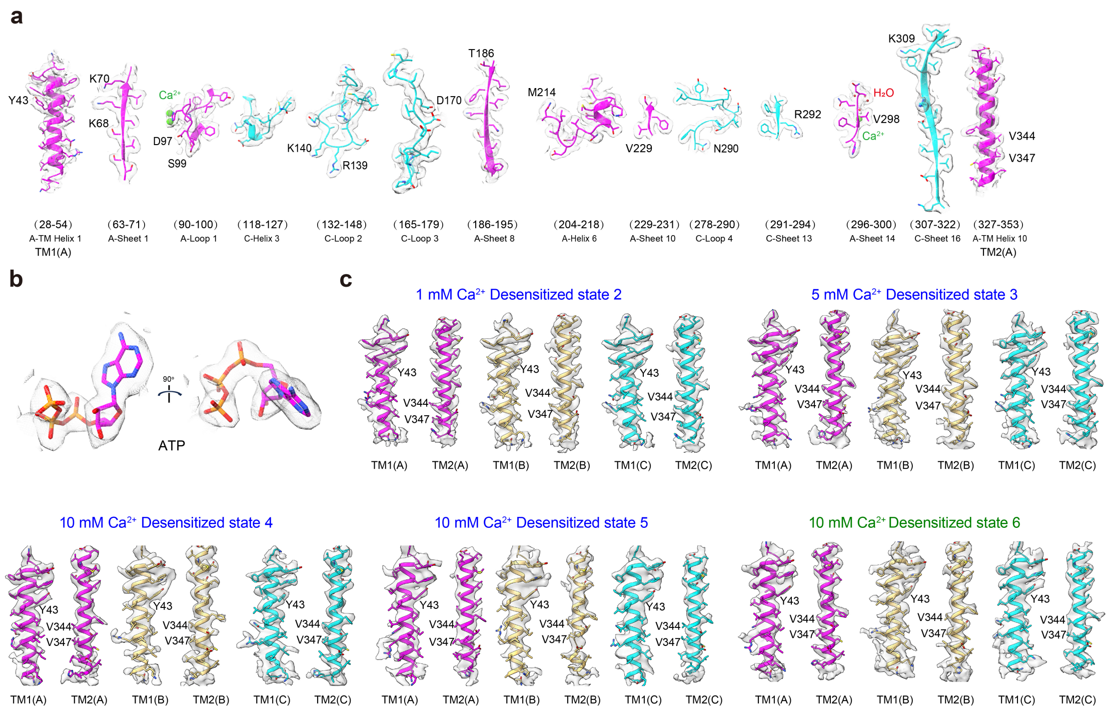

Supplementary Fig. S3 Quality of mP2X1 receptor cryo-EM maps. a, Representative cryo-EM densities of various structural elements in the mP2X1 receptor in 0 mM Calcium ions, the elements of protomer A colored in magenta, the elements of protomer C colored in Cyan, EM densities at a contour level of 0.30. **b,** Cryo-EM densities of ATP in the mP2X1 receptor in the desensitized state 1 at a contour level of 0.30. **c,** Transmembrane helices Cryo-EM densities of mP2X1 receptor in different concentrations of calcium ion.

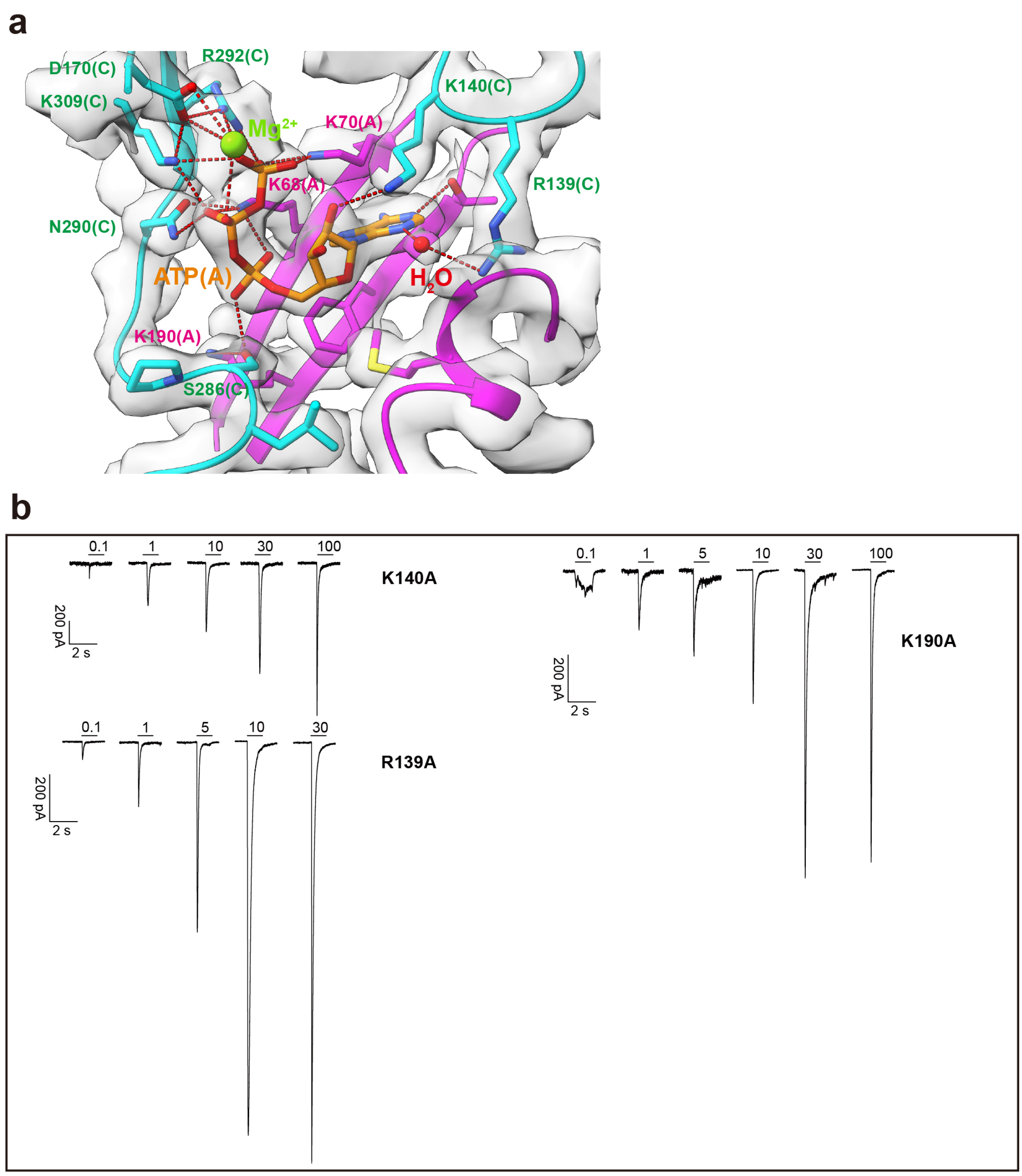

Supplementary Fig. S4 Polar interactions network of ATP and mP2X1 receptor. a, The structural model and density of ATP binding pocket of mP2X1 receptor, polar interactions shown in red dash line, density at contour level of 0.3. **b,** Representative currents of mP2X1 receptor R139A evoked by ATP (0.1-30 μM), mP2X1 receptor K140A evoked by ATP (0.1-100 μM), mP2X1 receptor K190A evoked by ATP (0.1–100 μM).

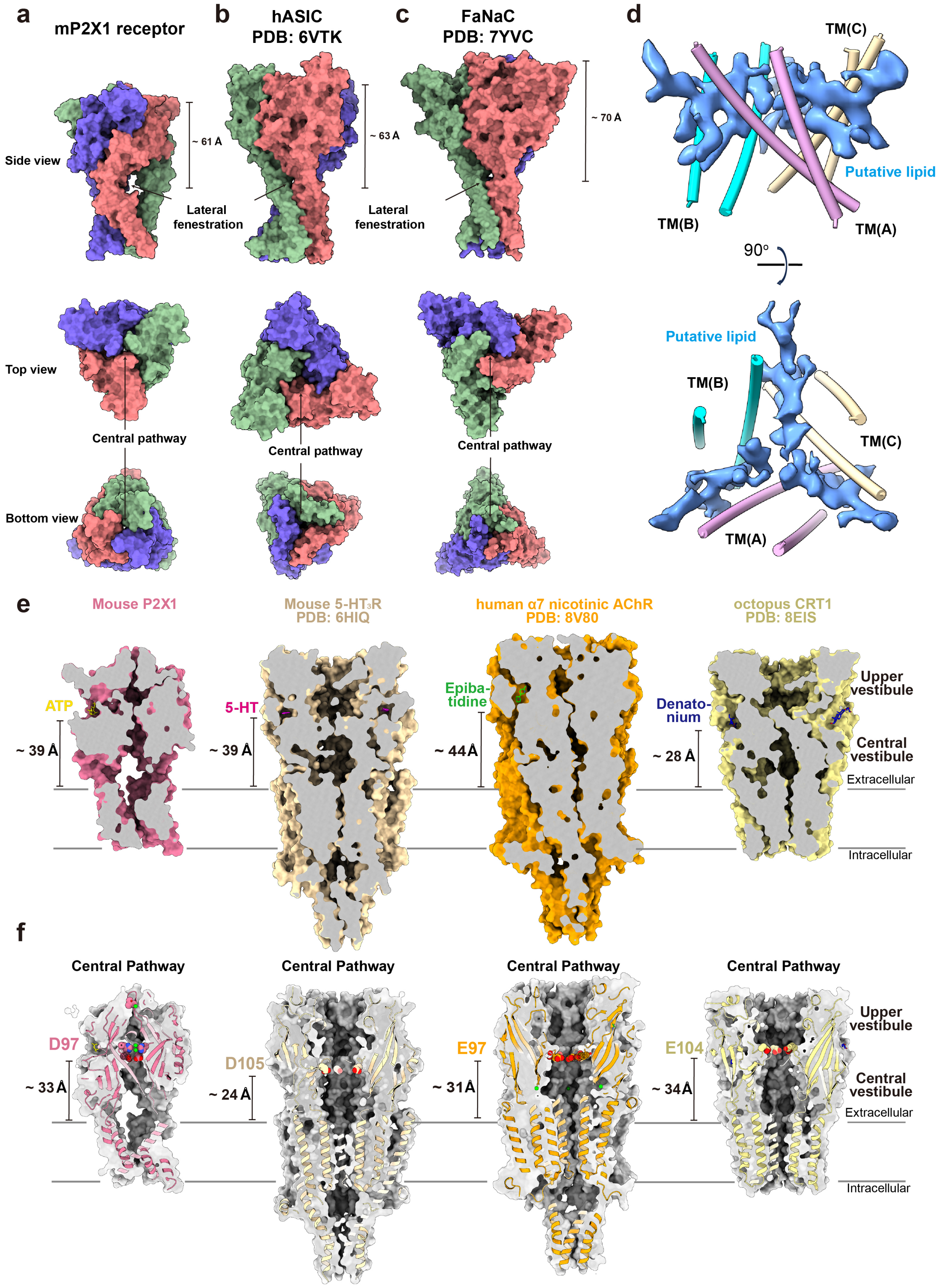

Supplementary Fig. S5 Unique features of the mouse P2X1. a, b, c, Views of parallel to the membrane (upper), from the perpendicular to the membrane from the extracellular surface (middle) and viewed from the perpendicular to the membrane from the intracellular surface (down) of mP2X1 (**a**), hASIC (**b**) and FaNaC (**c**). **d,** The putative lipids located in the two protomers' extracellular transmembrane interface and the extracellular vestibule. **e,** The distance between the calcium selectivity filter and the transmembrane domain is

similar across mouse P2X1 receptor, mouse $5HT_3R$, human α7 nicotinic AChR and octopus chemotactile receptor. **f,** The distances between the ligand-binding site and the transmembrane domain of mouse P2X1 receptor, mouse $5HT_3R$, human α7 nicotinic AChR and octopus chemotactile receptor.

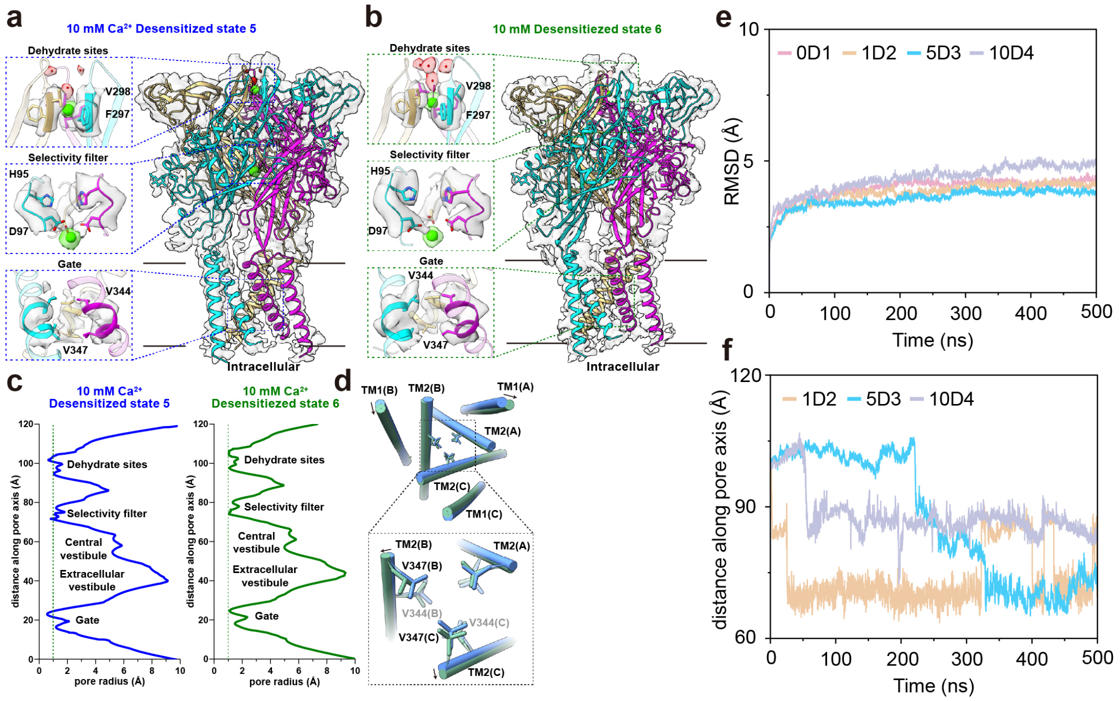

Supplementary Fig. S6 Structural analysis of P2X1 receptor in various concentration calcium ions. a, b, Structural model superimposition within the density map of the mouse P2X1 receptor in its 10 mM Ca^{2+} desensitized state 5 (**a**) and desensitized state 6 (**b**). The left panel displays, from top to bottom, the dehydration site, the selectivity site, the local gating model, and the corresponding density map. **c,** Radius of the central pathway, extending from the extracellular tip to the cytoplasmic side of the mouse P2X1 receptor in its 10 mM Ca^{2+} desensitized state 5 (left) and desensitized state 6 (right). **d,** Structural model superimposition of the mouse P2X1 receptor transmembrane domains in its 10 mM Ca^{2+} desensitized state 4 (blue) and desensitized state 6 (green). **e,** RMSD of P2X1 heavy atoms under different Ca^{2+} conditions during 500-ns molecular dynamics simulations for 0D1 (0 mM Ca^{2+}, desensitized state 1), 1D2 (1 mM Ca^{2+}, desensitized state 2), 5D3 (5 mM Ca^{2+}, desensitized state 3), and 10D4 (10 mM Ca^{2+}, desensitized state 4). **f,** Distance of Ca^{2+} ions along the central axis of the selectivity filter during 500-ns molecular dynamics simulations of 0D1, 1D2, 5D3, and 10D4.

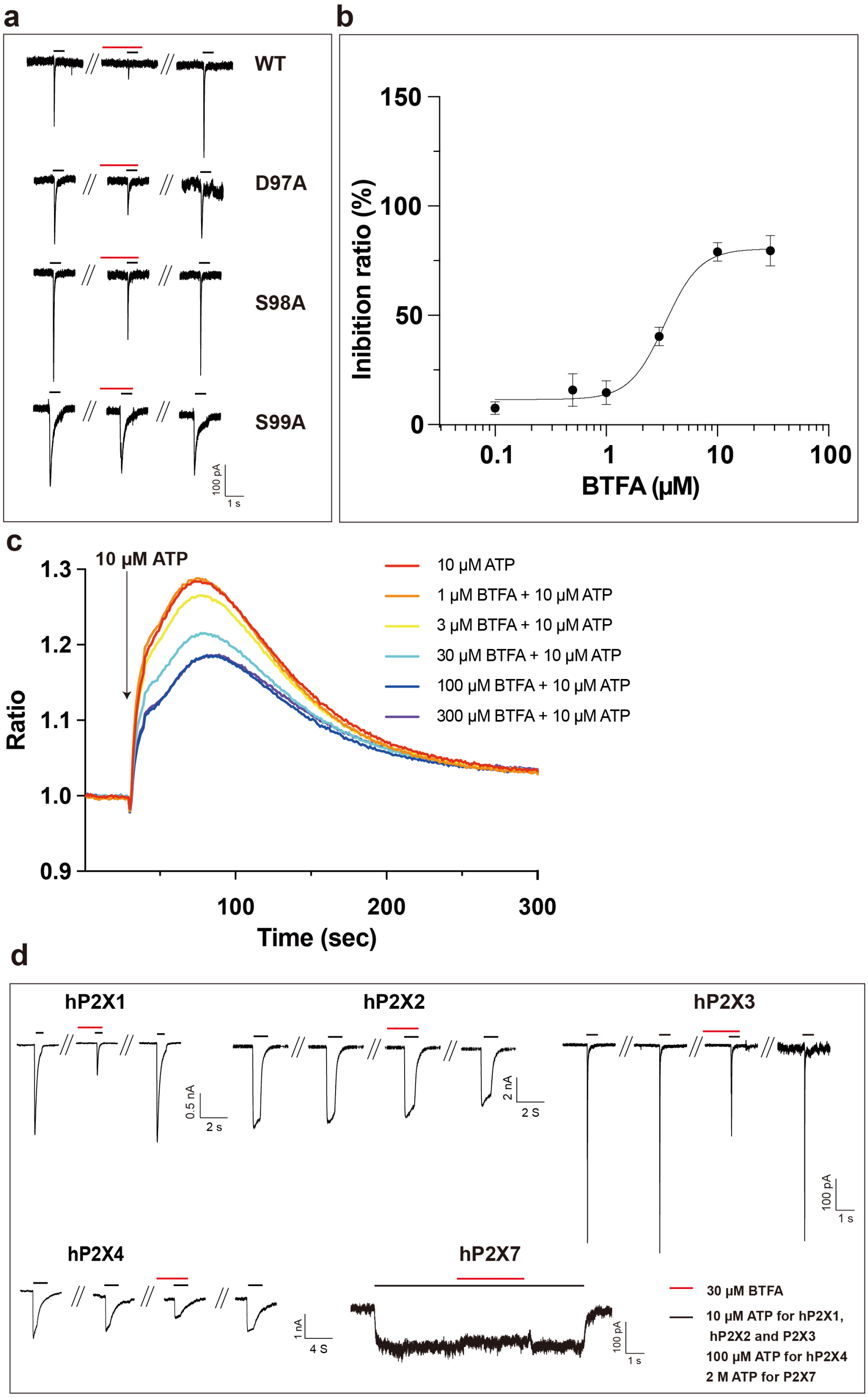

Supplementary Fig. S7 The top of central vestibule is an ion channel blocking site. **a,** Representative currents of BTFA binding pocket mutations of mP2X1 receptor evoked by 10 μM ATP. **b,** Dose–response curves of BTFA on mouse P2X1 receptor (10 μM ATP elicit). The estimated IC50 value was 4.4 ± 0.66 μM (n=9 for each tested BTFA concentration). **c,** Ca^{2+} influx in HEK293T cells transfected with P2X1 plasmids at various concentrations of BTFA (n = 6 samples). **d,** Inhibition of BTFA at human P2X receptor subtypes, the data presented as mean ± SEM, with n=5-10 independent cells examined over ≥3 independent experiments.

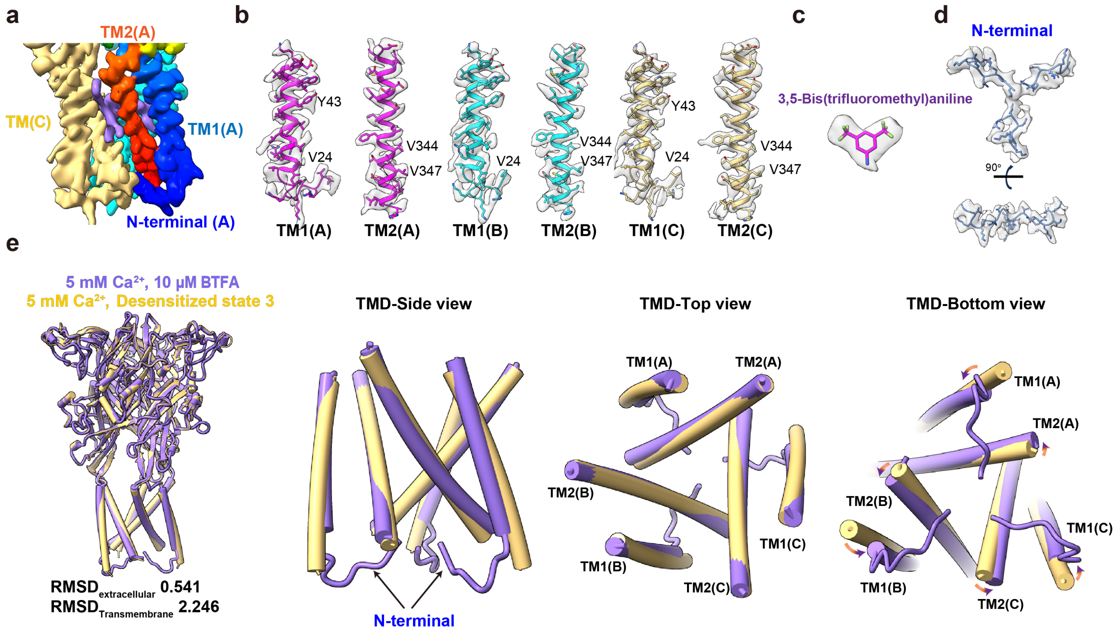

Supplementary Fig. S8 Structural comparisons of the mP2X1 receptor in 5 mM Calcium ions and 10 μM BTFA. a, b, Transmembrane helices Cryo-EM densities of mP2X1 receptor in 10 μM BTFA. **c,** Cryo-EM density of BTFA. **d,** Cryo-EM densities of N-terminal (V24–G30) of mP2X1.**e,** Overall and transmembrane domains structural comparisons of mP2X1 receptor in in 5 mM Calcium ions (colored in khaki) and 10 μM BTFA (colored in medium slate blue).

Supplementary Table S1: Data collection and structure refinement statistics.

	P2X1-Apo	P2X1-1 mM Ca^{2+}	P2X1-5 mM Ca^{2+}	P2X1-10 mM Ca^{2+}			P2X1-5 mM Ca^{2+} 10 μM BTFA
	Desensitized state 1	Desensitized state 2	Desensitized state 3	Desensitized state 4	Desensitized state 5	Desensitized state 6	
	PDB: 8ZT2	PDB: 8ZT5	PDB: 8ZT8	PDB: 8ZTA	PDB: 8ZTD	PDB: 8ZTF	PDB: 9XQR
	EMDB: 60445	EMDB: 60447	EMDB: 60453	EMDB: 60457	EMDB: 60458	EMDB: 60460	EMDB: 67120
**Data collection and processing**							
Magnification	105, 000						
Voltage (kV)	300						
Electron exposure (e-/Å^2)	50						
Defocus range (μm)	-1.0~-3.0						
Pixel size (Å)	0.832	0.81	0.73				
Symmetry imposed	C3	C3	C1	C1			C1
Initial particle images (no.)	1,324,707	1,118,210	1,792,965	6.038,471			8,925,734
Final particle images (no.)	200,491	245,073	144,342	31,517	43,811	192,582	218,684
Map resolution (Å)	2.31	2.32	2.34	2.58	2.63	2.43	3.14
FSC threshold	0.143	0.143	0.143	0.143	0.143	0.143	0.143
Map sharping B factor (Å^2)	-98.3	-94.6	-74.6	-67.9	-70.1	-83.1	-99.8
**Refinement**							
Initial mode used		P2X1-Apo	P2X1-Apo	P2X1-Apo	P2X1-Apo	P2X1-Apo	P2X1-Apo
Model resolution (Å)	2.41	2.45	2.38	2.61	2.67	2.47	3.50
FSC threshold	0.5	0.5	0.5	0.5	0.5	0.5	0.5
Model-Map CC (mask)	0.63	0.66	0.67	0.68	0.64	0.66	0.71
Model composition							
Protein residues	999	999	989	989	989	989	997
Waters	4	6	7	5	5	7	4
ATP	3	3	3	3	3	3	3
NAGs	9	9	9	9	9	9	9
ions	Ca^{2+}:5, Na^{+}:1	Ca^{2+}:3, Na^{+}:2, Mg^{2+}:3	Ca^{2+}:3, Mg^{2+}:3	Ca^{2+}:5, Mg^{2+}:3	Ca^{2+}:4, Mg^{2+}:3	Ca^{2+}:3, Mg^{2+}:3	Ca^{2+}:1, Mg^{2+}:3
BTFA							1
B factors (Å^2)							
Protein	26.46	26.46	26.46	26.46	26.46	26.46	26.50
Ligand	20.38	20.34	20.26	20.34	20.30	20.13	20.97
R.m.s.deviations							
Bond lengths	0.002	0.002	0.002	0.002	0.002	0.002	0.002
Bond angles	0.591	0.594	0.598	0.590	0.597	0.588	0.600
Validation							
MolProbity score	1.89	1.84	1.85	1.83	1.91	1.80	1.50
Clash score	7.23	7.92	7.54	7.98	7.23	7.10	8.27
Rotamer outliers (%)	2.15	3.77	3.77	3.30	4.72	3.42	1.17
Ramachandran plot							
Favored (%)	98.88	98.78	98.37	98.27	98.27	98.37	99.19
Allowed (%)	1.12	1.22	1.63	1.73	1.73	1.63	0.81
Disallowed (%)	0.00	0.00	0.00	0.00	0.00	0.00	0.00

Supplementary Video S1: Movie track of MD simulation trajectory of Ca^{2+} during 500-ns molecular dynamics simulations of 1D2.

Supplementary Video S2: Movie track of MD simulation trajectory of Ca^{2+} during 500-ns molecular dynamics simulations of 5D3.

Supplementary Video S3: Movie track of MD simulation trajectory of Ca^{2+} during 500-ns molecular dynamics simulations of 10D4.
